# Novel Marker for the Onset of Frontotemporal Dementia: Early Increase in Activity-Dependent Neuroprotective Protein (ADNP) in the Face of Tau Mutation

**DOI:** 10.1371/journal.pone.0087383

**Published:** 2014-01-29

**Authors:** Yulie Schirer, Anna Malishkevich, Yotam Ophir, Jada Lewis, Eliezer Giladi, Illana Gozes

**Affiliations:** 1 The Adams Super Center for Brain Studies, The Lily and Avraham Gildor Chair for the Investigation of Growth Factors, The Elton Laboratory for Neuroendocrinology, Department of Human Molecular Genetics and Biochemistry, Sackler Faculty of Medicine and Sagol School of Neuroscience, Tel Aviv University, Tel Aviv, Israel; 2 Center for Translational Research in Neurodegenerative Disease, University of Florida College of Medicine, Gainesville, Florida, United States of America; Centre Hospitalier de l'Université Laval, Canada

## Abstract

Tauopathy, a major pathology in Alzheimer's disease, is also found in ∼50% of frontotemporal dementias (FTDs). Tau transcript, a product of a single gene, undergoes alternative splicing to yield 6 protein species, each with either 3 or 4 microtubule binding repeat domains (tau 3R or 4R, associated with dynamic and stable microtubules, respectively). While the healthy human brain shows a 1/1 ratio of tau 3R/4R, this ratio may be dramatically changed in the FTD brain. We have previously discovered that activity-dependent neuroprotective protein (ADNP) is essential for brain formation in the mouse, with ADNP+/− mice exhibiting tauopathy, age-driven neurodegeneration and behavioral deficits. Here, in transgenic mice overexpressing a mutated tau 4R species, in the cerebral cortex but not in the cerebellum, we showed significantly increased ADNP expression (∼3-fold transcripts) in the cerebral cortex of young transgenic mice (∼disease onset), but not in the cerebellum, as compared to control littermates. The transgene-age-related increased ADNP expression paralleled augmented dynamic tau 3R transcript level compared to control littermates. Blocking mutated tau 4R transgene expression resulted in normalization of ADNP and tau 3R expression. ADNP was previously shown to be a member of the SWItch/Sucrose NonFermentable (SWI/SNF) chromatin remodeling complex. Here, Brahma (Brm), a component of the SWI/SNF complex regulating alternative splicing, showed a similar developmental expression pattern to ADNP. Immunoprecipitations further suggested Brm-ADNP interaction coupled to ADNP - polypyrimidine tract-binding protein (PTB)-associated splicing factor (PSF)-binding, with PSF being a direct regulator of tau transcript splicing. It should be noted that although we have shown a correlation between levels of ADNP and tau isoform expression three months of age, we are not presenting evidence of a direct link between the two. Future research into ADNP/tau relations is warranted.

## Introduction

Activity-dependent neuroprotective protein (ADNP) [1], [2], [3], a protein responsive to brain injury [4], [5], [6] is essential for brain formation [7]. ADNP's binding partners include heterochromatin protein 1 (HP1) [8], [9] interacting with the SWI/SNF chromatin remodeling complex [8], [10], which is associated with transcription and splicing [11]. While complete ADNP deficiency is lethal, ADNP heterozygous mice (ADNP^+/−^) exhibited cognitive deficits, significant increase in phosphorylated tau, tangle-like structures (tauopathy), reduced neuronal survival and neurodegeneration [12].

Neurodegenerations, including Alzheimer's disease (AD) and frontotemporal dementia (FTD) are characterized by tauopathy. Tau plays a central role in the promotion of microtubule (MT) assembly and stabilization of the MT network, allowing normal axonal growth and axonal transport [13], [14]. There are six major isoforms of tau in the adult human brain, all of which are derived from a single gene, on the long arm of chromosome 17, by alternative splicing [15], [16], [17]. Tau is characterized by the presence of a MT binding domain, which is composed of 3 or 4 repeats (3R and 4R tau) of a highly conserved tubulin binding motif (exon 10 on the tau gene encodes the additional tubulin binding site in the 4R tau). This domain comprises the carboxy terminal (C-terminal) half of the protein, followed by a basic proline-rich region and an acidic amino-terminal (N-terminal) region, which is normally referred to as the ‘projection domain’. The six tau isoforms differ from each other in the number of tubulin-binding repeats (3R and 4R tau isoforms) and in the presence or absence of either one or two 29 amino-acid long inserts at the N-terminal portion of the protein, which is not instrumental for MT binding. The various isoforms appear to be differentially expressed during development, however the 3R and 4R tau isoforms are expressed in a 1:1 ratio in most regions of the adult human brain, and deviations from this ratio are characteristic of FTD tauopathies [18]. Interestingly, recent findings identified stress-induced 3R tau localization to the nucleus, providing protection against DNA damage [19], while ectopic overexpression of full length tau altered the nuclear architecture [20] and changed the cellular localization of tau splicing proteins [21]. Tau hyperphosphorylation, is associated with a loss of MT binding capacity [22] and is considered to be a central element in the pathogenesis of AD and FTD. Neuronal loss was linked to the topographic distribution of neurofibrillary tangles in several stereological studies in AD brains [23]. Mutations in the tau gene have been identified in families suffering from hereditary FTD and Parkinsonism linked to chromosome 17 (FTDP-17) [24], [25].

An example of a mouse tauopathy model encompassing brain region specific tau hyperphosphorylation, tangle-like formation, neuronal cell death and behavioral defects is the rTg(tauP301L)4510, generated using a system of responder and activator transgenes as described [26], [27]. Here, we have hypothesized that ADNP expression 1] is regulated by the expression of FTD-related mutated tau, and 2] by regulating tau transcript splicing, provides for reciprocal relationship and tight control of neuronal homeostasis. The hypothesis was tested using 1] the rTg(tauP301L)4510 mouse and 2] co-immunoprecipitation with splicing protein factors.

## Materials and Methods

### Animals

The mouse model rTg(tauP301L)4510 used in the current study was under material transfer agreement (MTA) from the Mayo Clinic (Jacksonville, Florida) with Allon Therapeutics Inc. and was maintained as before [26], [27]. Protocols were approved by the Israeli Ministry of Health and the Tel Aviv University Committee for Animal Welfare in Research.

Mouse genotypes were obtained by polymerase chain reaction (PCR) analysis of genomic DNA extracted from the tail. The primer pairs that were used are 5′-GATTAACAGCGCATTAGAGCTG-3′ and 5′-GCATATGATCAATTCAAGGCCGATAAG-3′ for activator transgenes and 5′-TGAACCAGGATGGCTGAGCC-3′ and 5′-TTGTCATCGCTTCCAGTCCCCG-3′ for responder transgenes. Mouse T-cell receptor was use as a positive control 5′-CAAATGTTGCTTGTCTGGTG-3′ and 5′-GTCAGTCGAGTGCACAGTTT-3′. Mice carrying both responder and activator transgenes henceforth called transgenic (Tau-Tg) and mice carrying a single transgene (tau responder) served as controls non-tau transgenic called here in short non-transgenic (non-Tg). For rapid genotyping we used the services of Transnetyx Inc., Cordova, TN, USA.

The experiment included five groups of Tau-Tg male mice, 1-, 3-, 5.5-, 9- and 10.5-month-old (n = 20), and as control, non-Tg male mice at the same ages (n = 20). Five mice/age-group were analyzed for cortical ADNP expression and 4 mice/age-group were analyzed for cerebellar ADNP expression. For doxycycline inhibition experiments, 2-month-old mice were used and doxycycline (Sigma-Aldrich) was administrated in the drinking water for three weeks, 5 mice were used per group [26], [27].

Mice were sacrificed by CO_2_ inhalation, their brains removed and dissected into cortex and cerebellum and kept frozen at −80°C for further analysis.

A second mouse model used in the current study was the ADNP haploinsufficient mouse model (ADNP+/−) [12], with outbreeding with ICR mice. Here, female mice (n = 4–5/group) were used and were sacrificed by cervical dislocation. Cerebral cortical tissues were removed and kept frozen at −80°C for further analysis.

Additional animals used were newborn rats for control brain extract preparation, as before [28].

### Biochemical and immunochemical procedures

Total levels of nuclear ADNP expression were assessed by western blot analysis using actin as a control [29]. Cerebral cortex samples (∼50 mg each) and cerebellum samples (∼50 mg each) were homogenized and separated to cytosolic and nuclear fractions according to published literature [30] with slight modifications. Homogenized samples separated to cytoplasmic fractions used a lysis buffer (20 mM Tris-HCl pH 7.7, 10 mM KCl, 0.1 mM EDTA, 1.5 mM MgCl2, 0.2% IGEPAL) and included incubation on ice for 5 minutes and centrifugation at 3200 g for 5 min, with the supernatant yielding the desired cellular fraction. The pellet resulting from the centrifugation above was further subjected to extraction buffer (10 mM Tris HCl pH 7.7, 0.1 mM EDTA, 1.5 mM MgCl2, 20% glycerol, 550 mM NaCl), followed by incubation on ice for 30 minutes and centrifugation at 17,530 g for 15 min. Protein amount was estimated and corrected by the BCA-200 protein assay kit (Pierce, Rockford, IL, USA) and equal protein amounts were loaded onto 10% polyacrylamide gels containing SDS (20 µg/lane), [2]. Western blot analyses were performed by applying brain protein samples onto two gels; one for transgenic mice and one for control, non-transgenic, brain samples. Each gel had sample representation from four of the five age groups; cerebral cortex sample gels included brain samples of mice aged 3-, 5.5-, 9- and 10.5-months. Cerebellum sample gels included brain samples of mice aged 1-, 3-, 5.5- and 9-months.

The proteins were transferred to nitrocellulose filter (Millipore, Bedford, MA) and immunostained with specific antibodies directed against ADNP at 150KD: A mouse monoclonal antibody (1∶300 dilution or as indicated; BD Bioscience, Franklin Lakes, NJ, USA) was used as well as a rabbit polyclonal antibody (1∶500 dilution; Bethyl Laboratories, Montgomery, TX, USA). The epitope recognized by the antibodies maps to a region between residues 1050–1102 C-terminal region of ADNP [2]. Secondary antibodies conjugated horse radish peroxidase (HRP) recognizing rabbit or mouse immunoglobulins were used (1∶5000; Jackson Immunoresearch, West Grove, PA, USA). Proteins were visualized using enhanced chemiluminescence (ECL) reagents (Pierce, Rockford, IL, USA) followed by exposure onto hyperfilm (Kodak, Petach Tikva, Israel). Protein bands on hyperfilm were quantified using photochromatography analysis (DNR Bio-Imaging Systems Ltd. Maale Hachamisha, Israel). The ADNP amount in each band was calculated as its percentage from the total amount of all bands, and was then divided by the correlating actin amount of the same sample [29]. ADNP/actin ratios of each group were averaged.

Cytosolic tau-3R expression was detected using monoclonal mouse tau (3-repeat isoform RD3) antibody (1∶1000; Millipore, Billerica, MA, USA) and (human) htau-4R (4-repeat isoform RD4), clone 1E1/A6 antibody (1∶1000; Millipore, Billerica, MA, USA). Primary antibody binding was detected and quantified as above with actin as a control (above) and Brahma (Brm) as a second control [hBrm/hSNF2α (1∶1000; Sigma, Rehovot, Israel].

### Quantitative real time RT-PCR

ADNP and tau3R mRNA levels of Tg mice (1, 3, 5.5, 9 month-old) were obtained by quantitative real time RT-PCR. RNA was isolated using MasterPure™ RNA Purification Kit (Epicentre Biotechnologies, Madison, WI, USA). RNA concentration was determined by Nanodrop ND-1000 UV-Vis spectrophotometer (Nanodrop Technology, Wilmington, DL, USA). Samples containing equal amount of total RNA (0.2–1 µg RNA/sample) were subjected to reverse transcription (RT) using High-capacity cDNA reverse transcription kit (Applied Biosystems, Foster City, CA, USA). Real Time PCR was performed using Powered SYBR Green PCR master kit (Life Technologies, Applied Biosystems, Carlsbad, CA, USA) and ABI PRISM 7900 Sequence Detection System instrument and software (Applied Biosystems). RNA expression levels were determined using specific primers for mouse ADNP (NM_009628) 5′- ACGAAAAATCAGGACTATCGG-3′ and 5′-GGACATTCCGGAAATGACTTT-3′, mouse tau 3R and mouse tau 4R RNA were determined using the following the primers. Tau 3R sense: 5′- TGGCAAGGTGCAAATAGTCT-3′; antisense: 5′-TGGCTTGTGATGGATGTTCC-3′; tau 4R sense: 5′-GTGGCAAGGTGCAGATAAT-3′ antisense: 5′-CCGGGACGTGTTTGATATTA-3′. HPRT (hypoxanthine-guanine phosphoribosyltransferase) was used as the normalization control gene; Mouse HPRT1 (NM_013556) 5′-GGATTTGAATCACGTTTGTGTC-3′ and 5′-CAGGACTCCTCGTATTTGCAG-3′.

### Immunoprecipitation

Cytoplasmic and nuclear proteins from male C57BL/129 wild type, 11-month-old mouse brains were separated as above. Protein amount was estimated by using the Bradford protein assay (Biorad, Hercules, CA, USA). Proteins (400–500 µg) were taken from the nuclear-enriched fraction of the brain without cerebellum, cortex and hippocampus (i.e. thalamus and subthalamic nucleus) and diluted in extraction buffer with reduced concentration of salt (150 mM NaCl) for further immunoprecipiations. The proteins were incubated with 2–5 µg rabbit anti-hBrm/hSNF2α (Sigma, Rehovot, Israel) at 11 rpm, program F5 (Rottamix RM1, ELMI, Riga, LV) at 4°C. Protein A/G PLUS-Agarose beads (Santa Cruz Biotechnology, Santa Cruz, CA, USA) were then added to each fraction followed by an additional 3 hour-incubation at 11 rpm, program F5 (Rottamix RM1, ELMI, as above) at 4°C. Following the incubation periods above, brain fractions were subjected to centrifugation at 2000 rpm (Eppendorf centrifuge 5417R, Hauppauge, NY, USA) for 2 minutes and the supernatants were discarded. To remove unbound non-specific proteins, the pellet was washed with 1 ml of cold extraction buffer (150 Mm NaCl) and subjected to 2000 rpm centrifugation for 2 minutes, then washed (X2) with 1 ml of cold PBS pH 7.4 (Biological Industries, Beit-HaEmek, Israel) containing complete protease inhibitor cocktail tablets, EDTA-free (1∶25, Roche, Indianapolis, IN, USA), and subjected to centrifugation at 2000 rpm (as above) for 1 minute. The dry pellet of the protein-bead complex was diluted in 30 µl of sample buffer X2 (10% SDS, 15% β-mercaptoethanol, 10% glycerol, 0.005% bromophenol blue, 0.5 M Tris HCl) pH 6.8. To exclude the proteins from the beads, the samples were boiled at 100°C for 5 minutes. The proteins were then separated by electrophoresis on 8% polyacrylamide gel containing SDS. The separated proteins were transferred to nitrocellulose filters and immunostained with rabbit polyclonal antibody against hBrm/hSNF2α at 230 KD (above) and rabbit polyclonal antibody against ADNP 150 KD (1∶500 dilution; Bethyl Laboratories, above). Immunoreactive protein bands were visualized as above.

A second protein analyzed for co-immunoprecipitation with ADNP was PSF [polypyrimidine-tract binding protein (PTB) -associated splicing factor], using the mouse anti-PSF (Sigma, Rehovot, Israel). Proteins (500 µg) were taken from the nuclear-enriched fraction of the cerebellum from 8-month-old C57BL/129 male mice and diluted in extraction buffer with reduced concentration of salt (150 mM NaCl) for further immunoprecipitation using the Co-IP-cross-link kit (Pierce) protocol as follows. 20 µl of A/G PLUS-Agarose beads were loaded to the column washed with coupling buffer at 1000 rpm for 1 minute. 10 µg of mouse PSF antibody were added to the beads and following one hour incubation at 24°C were cross-linked using 2.5 mM disuccinimidyl suberate (DSS) for an additional hour. After that, cleared 500 µg of brain lysate samples were added to the column and incubated (16 hours, 4°C). To detect the eluted antigen, western blot analysis was performed with the Co-IP kit sample loading buffer which was used under non-reducing conditions, followed by 10% polyacrylamide gel electrophoresis for PSF detection and 8% for ADNP detection.

A third series of immunoprecipitation experiments included 3-month-old male mice comparing ADNP expressing mice (ADNP+/+) and ADNP haploinsufficient mice (ADNP+/−). The NE-PER kit (Thermo, Hudson, New Hampshire USA) was used to separate nuclear and cytoplasmic fractions from cortical extracts. 500 µg nuclear cerebral cortex proteins were used for further IP procedure with the Co-IP-cross-link kit as above using ADNP, PSF and Brm antibodies. Western analysis was performed as above.

### Statistical analyses

Results are described as means ± standard error of the mean (S.E.M). Statistical analyses were performed using Factorial ANOVA followed by Tukey's Honestly Significantly Different (HSD) post-hoc test or Student-Newman-Keuls Method, as indicated. P values of 0.05 were deemed statistically significant. When needed, one way ANOVA was performed with the appropriate post-hoc test as indicated below. When only two groups were compared, the Student's t test was utilized.

## Results

### ADNP expression in the cerebral cortex is correlated with tau expression and disease onset

**Figure 1:**
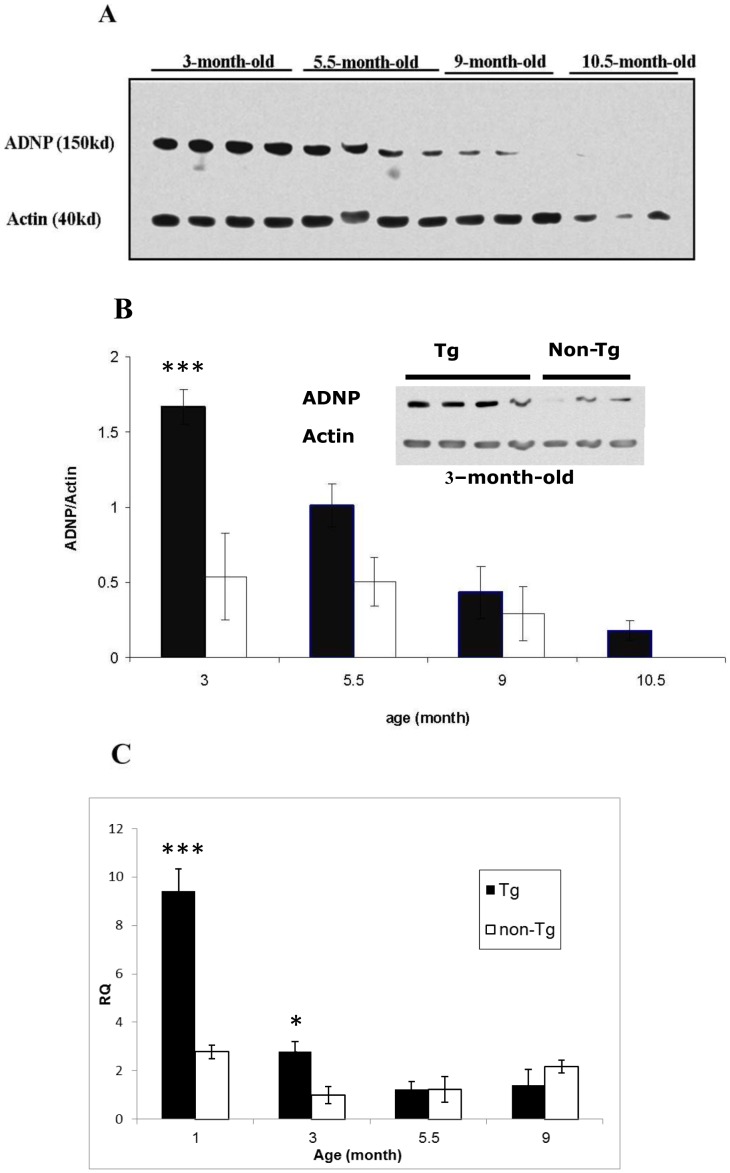
Age-dependent changes in the expression of ADNP: Differences between Tau-Tg and control mice. A] Tau-Tg mice western blot analyses. Western blot analyses for ADNP were performed on 3-10.5-month-old-mice (3 or 4 replicates/age group, with 4–5 mice/group). One blot is shown, including extracts from 3-10.5-month-old-mice with actin as a control protein (each lane represents 1 mouse and 4 mice are shown per group). B] Age-dependent changes in the expression of ADNP at the protein level in the cerebral cortex: Differences between Tau-Tg and control mice. The ADNP/actin ratio in each immunoreactive band (in A) was calculated as the ADNP percentage from the total amount of all bands, and then divided by the correlating actin amount of the same sample. ADNP/actin ratios for each group were averaged (n = 5/group, average of 3–4 gel replicates). Quantitative densitometry is shown for four ages: 3, 5.5, 9 and 10.5 months (black bars, Tau-Tg; white bars, littermates not expressing the 4R mutated tau as outlined in the method section, *** *p*<0.001, Tau-Tg vs. control mice, see results section). The 10.5-month point shows only the Tau-Tg ADNP expression. Additional experiments [54] compared Tau-Tg and controls revealing no differences at the actin level (densitometry results of 8.4±3.4, vs. 8.3±2.6). In contrast, ADNP levels were significantly decreased in the Tau-Tg 5.5±0.9, vs. controls 11.2±1.35, *p*<0.01 [54]. The insert is showing a western blot comparing ADNP expression in Tau-Tg (n = 4) and controls (n = 3), at the age of 3 months, each lane represents 1 mouse. C] Age-dependent changes in the expression of ADNP mRNA in the cerebral cortex: Differences between Tau-Tg and control mice. The ADNP mRNA amount in each sample was calculated using the correlated HPRT mRNA amount of the same sample. ADNP mRNA amounts for each group were averaged. [Tg: 1-month-old 9.422+0.92, 3-month-old 2.788±0.407, 5.5-month-old 1.242±0.314, 9-month-old 1.402±0.642. Non-Tg: 1-month-old 2.78+0.28, 3-month-old 1±0.357, 5.5-month-old 1.222±0.535, 9-month-old 2.18±0.27, n = 5/age group]. RQ  =  relative quantity (http://de-de.invitrogen.com/etc/medialib/en/filelibrary/Nucleic-Acid-Amplification-Expression-Profiling/PDFs.Par.83765.File.dat/relative-quant-ct.pdf), (*** *p*<0.01).

Nuclear protein extracts from the cerebral cortex of Tau-Tg mice were subjected to western blot analysis with ADNP and actin antibodies (Fig. 1A) coupled to quantitative densitometry for ADNP/actin ratios (Fig. 1B). Protein samples at ages 3, 5.5, 9 and 10.5 months were compared. Results indicated significantly high ADNP/actin ratio at 3 months of age in the Tau-Tg mice which continuously decreased by ∼30–50% between each evaluated time point (Fig. 1A-B). Comparison of the Tau-Tg with control littermates by one-way ANOVA analysis followed by All Pairwise Multiple Comparison Procedures (Student-Newman-Keuls Method) revealed main effects for age and genotype (DF-6, F- 15.126, *p*<0.001), with a significant ∼3-fold increase at 3-months in the Tau-Tg compared to the non-Tg (*p*<0.001), (Fig. 1B, the insert shows the western blot picture, at 3-months of age).

ADNP mRNA levels in the cerebral cortex were quantified by Real Time PCR using the correlated HPRT mRNA amount of the same sample (Fig. 1C). Results indicated increased ADNP mRNA expression at 1 and 3 months of age (∼5 and 3-fold, respectively) compared to control non-Tg mice (Fig. 1C, ANOVA analysis followed by Tukey's Multiple Comparison Test, *p*<0.01), which paralleled the increase of the ADNP/actin ratio at the protein level (Fig. 1B). Like the protein, ADNP mRNA decreased at 5.5 months of age leveling with control values (Fig. 1C), although at the protein level (nuclear protein extract), there seemed to be a trend for increased expression of ADNP/actin ratio even at 5.5 months in the Tau-Tg mice compared to non-Tg, this trend was insignificant (Fig. 1B).

### Doxycycline administration reduces ADNP expression in 3-month-old Tau-transgenic mice in correlation with tau 3R expression

**Figure 2:**
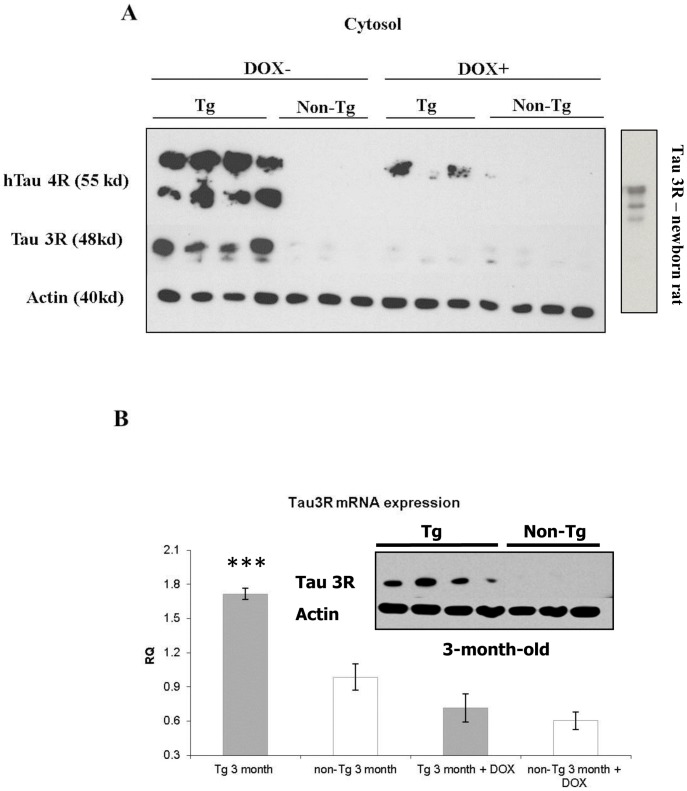
Doxycycline treatment shuts down transgene expression. A] Tau protein expression. Western blot analyses for the transgene expression human 4R tau (hTau 4R), mouse tau 3R and actin (as a control) were performed on cytoplasmic extracts prepared from the cerebral cortex tissues of control (Non-Tg) and transgenic mice (Tg) exposed to doxycycline (Dox+) treatment or littermates not exposed to doxycycline (Dox-). The insert shows a control tau 3R expression in newborn rat brain. B] Tau mRNA quantification. The tau mRNA amount in each sample was calculated using the correlated HPRT mRNA amount of the same sample. A comparison of Tg and non-Tg as well as Dox+ treatment is shown as for the proteins (panel A, above), (*** *p*<0.005).

Transgene expression in the rTg(tauP301L)4510 mouse model is induced via the tetracycline-operon responsive element and is suppressed after treatment with doxycycline. Two-month-old Tau-Tg (n = 4) and non-Tg (n = 3) mice were treated with doxycycline (Sigma-Aldrich). Doxycycline was administrated in the drinking water for three weeks. At the end of treatment period, mouse brains were removed and dissected. To verify doxycycline efficient reduction of transgene expression, tau 4R and 3R antibodies were used to monitor cytoplasmic tau expression in the cerebral cortex of the mice. Fig. 2 shows a dramatic reduction in human tau 4R (hTau 4R) expression as a consequence of doxycycline treatment, which is expected in this mouse model of controlled transgene expression [27]. In Fig. 2A, the hTau 4R antibody did not detect mouse 4R Tau, but showed some incomplete reduction in transgene expression in the doxycycline-treated mice. Tau 3R levels were also monitored showing a dramatic increase in the Tau-Tg animals compared to non-Tg mice of the same age (3 months) and a marked reduction upon doxycyline treatment to the non-Tg levels (Fig. 2A). The antibodies were also used with newborn control rat brain extracts [28], showing, as expected, 3R Tau immunoreactivity (Fig. 2A, insert). These results are in agreement with previous data, showing tau 3R over expression (slower turnover) in the tau 4R transgenic mice [26]. This data was further verified by monitoring tau 3R mRNA levels using quantitative real time PCR in the cortex of Tg-Tau and non-Tg mice, with and without doxycycline treatment. Results showed ∼2-fold increase in the tau 3R mRNA transcript in the Tg-Tau compared to the non-Tg in 3-month-old mice, which was significantly decreased (∼3-fold) to non-Tg levels following doxycycline treatment. One way ANOVA indicated significant differences (DF-3, F-12.217, *p*<0.001), All Pairwise Multiple Comparison Procedures (Student-Newman-Keuls Method) between the Tg-Tau and non-Tg mice (*p*<0.005) and a significant reversal following doxycyline treatment (Fig. 2B).

For comparison with ADNP relative expression, tau 3R mRNA expression was also assessed at the age of 5.5 months showing ∼3 fold age-dependent decrease (compared to 3 months of age) and no difference between Tau-Tg and non-Tg (data not shown). The changes (age and genotype) in tau 3R mRNA expression paralleled the changes in ADNP expression in the Tau-Tg mice, i.e. increased expression at 3 months and no significant differences at 5.5 months, when comparing Tau-Tg to non-Tg (Fig. 1).

**Figure 3:**
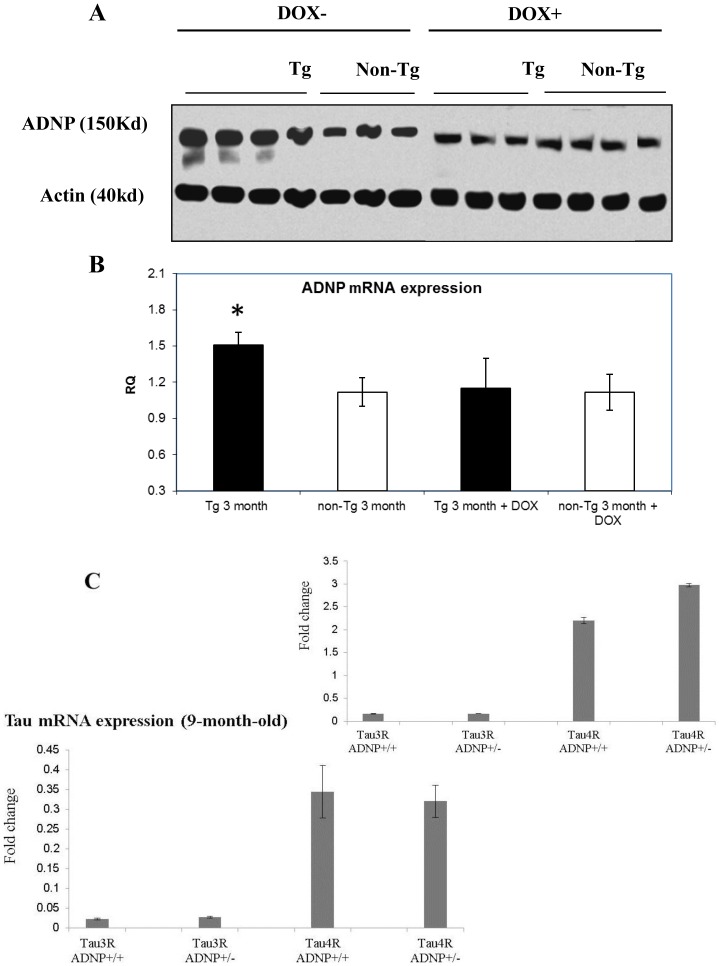
Doxycycline treatment which shuts down transgene expression normalizes ADNP and tau 3R expression, while ADNP deficiency may regulate tau isotype expression. A] ADNP expression with doxycycline: Western blot analysis is depicted as in Fig. 2A, on nuclear extracts with ADNP antibodies. When the ADNP/actin ratios were calculated as in Fig. 1, a significant reduction of ADNP/actin ratio was observed following doxycycline treatment, correlating ADNP expression to tau transgene expression (*p<0.05). Five mice were used per an experimental group; each gel lane includes one mouse extract with 3-4 mice representing an experimental group. B] ADNP mRNA quantification. The ADNP mRNA amount in each sample (n = 5/experimental group) was calculated using the correlated HPRT mRNA amount of the same sample. A comparison of Tg and non-Tg as well as Dox+ treatment is shown as for the proteins (panel A, above), (* *p*<0.05). C] Trend toward deregulation of tau expression in the cortex of 2-month-old ADNP+/− mice. The expression of mouse tau 3R and mouse tau 4R (4–5 female mice, cerebral cortex/experimental group) was compared by quantitative real time PCR and results are depicted on the graph, comparing 2-month-old to 9-month-old. There was a significant difference (P<0.05, one tailed, Student's t-test) in tau 4R expression in the 2-month-old ADNP+/- mice compared to ADNP+/+ mice. Data is presented as fold-change (2^−Δ*C*^_T_), [55].

Given the parallelism between ADNP mRNA expression and tau 3R expression (comparing Tau-Tg to non-Tg) as well as the change observed in tau 3R expression following docycylcline human mutated tau 4R shut down, ADNP expression was further evaluated following doxycycline treatment. Nuclear ADNP/actin ratios in the cerebral cortex were quantified by western blot analysis with ADNP and actin specific antibodies as above (Fig. 1). While untreated Tau-Tg mice showed increased ADNP expression in comparison to the non-Tg mice at the age of 3 months, Tau-Tg mice that were treated with doxycycline expressed similar ADNP levels as those expressed by the control non-Tg mice (Fig. 3A). One way ANOVA (DF-3, F-3.347, *p*<0.05) followed by All Pairwise Multiple Comparison Procedures (Student-Newman-Keuls Method), comparing the ADNP/actin ratios, before and after doxycycline treatment, showed a significant reduction in the Tau-tg mice, toward normalization at the non-Tg levels (p<0.05, Tau-Tg vs. doxycycline treated, Tau-Tg). Similar to the developmental changes, the protein results paralleled the changes in the respective ADNP mRNA (Fig. 3B), which were significantly decreased following doxycycline treatment to the non-Tg levels at 3 months of age. Furthermore, the ADNP normalization paralleled the normalization in tau 4R and 3R expression (Fig. 2).

As the results showing deregulation of ADNP expression in a tauopathy model paralleling endogenous tau 3R expression, and given our original data showing tau hyperphosphorylation and tangle-like formation in ADNP haploinsufficient mice (ADNP+/−) [12], we investigated if tau expression is deregulated in the cerebral cortex of ADNP+/− mice. Results (Fig. 3C) showed a trend toward increased tau 4R expression (exon inclusion) in 2-month-old ADNP+/− mice compared to ADNP+/+ controls (*p*<0.05, one tail, t-test comparing only tau 4R expression), which disappeared in older, 9-month-old mice, showing similar tau species expression in the ADNP+/+ and ADNP+/− mice (Fig. 3C).

### Nuclear ADNP expression in the cerebellum is not correlated with tauopathy, but is correlated with age

**Figure 4:**
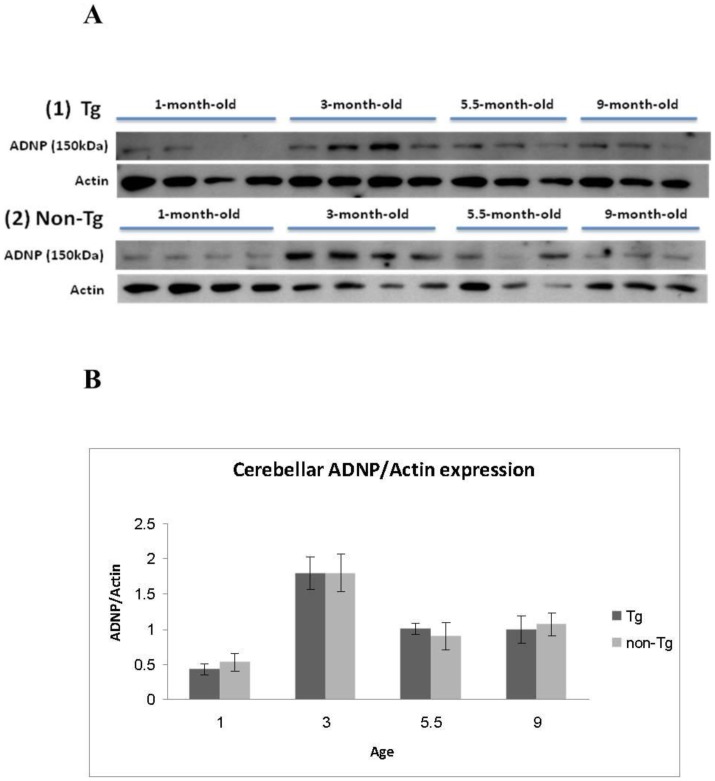
ADNP expression in the cerebellum is not subject to regulation by transgene expression in other brain areas. A] Western blot analysis. Expression of nuclear ADNP in the cerebellum of Tau-Tg mice (1) and non-Tg mice (2) is shown by gel electrophoresis and western blot analysis coupled to quantitative densitometry. Results show an increase in ADNP expression at 3 months of age in both transgenic and non-transgenic mice, followed by a decrease at 5.5 and 9 months of age (each experimental group included 4 mice, 3–4 representatives are shown). B] ADNP quantification in the cerebellum. The ADNP amount in each band was calculated as its percentage from the total amount of all bands, and was then divided by the correlated actin amount of the same sample. ADNP/actin amounts of each group were averaged [Tg: 1-month-old 0.43+0.078, 3-month- old 1.794+0.228, 5.5-month-old 1.009+0.08, 9-month-old 0.998+0.195. Non-Tg: 1-month-old 0.532+0.125, 3-month-old 1.798+0.264, 5.5-month-old 0.903+0.196, 9-month-old 0.406+0.166]. Four mice were used for each experimental point and gel electrophoresis was repeated twice, average results are shown. Statistical analysis is shown in the text.

**Figure 5:**
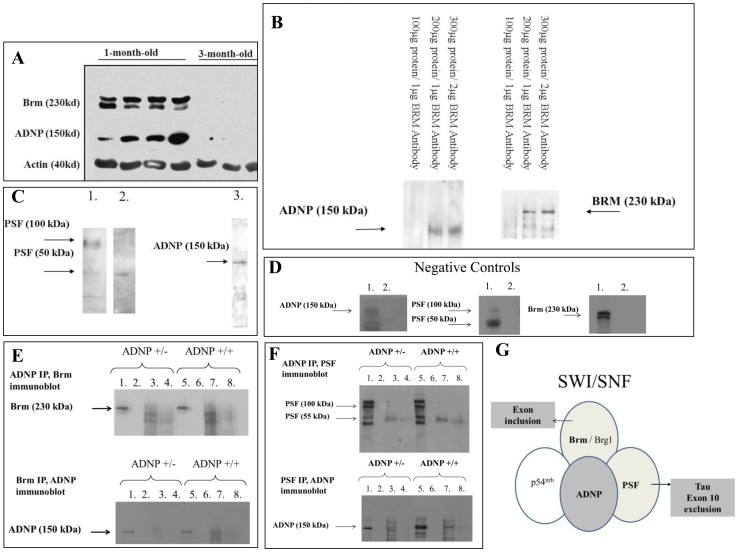
ADNP co-immunoprecipitates with splicing factors. A] Tau-Tg mice western blot analyses. Western blot analyses for Brm and ADNP were performed on 1- and 3-month-old-mice (3 or 4 replicates/age group, n = 5). One blot is shown, using actin as a control protein (results from 3–4 mice/experimental group are shown). B] Brm. Immunoprecipitation, gel electrophoresis (8% polyacrylamide) and western blot analysis was carried out as described in the method section. Increasing concentrations of nuclear protein extracts from the thalamus and subthalamic nucleus of 11-month-old C57BL/129 male mice were subjected to immunoprecipitation with Brm antibody (Sigma), 8% polyacrylamide gel electrophoresis and immunoblotting with either ADNP (BD) (lanes 1–3) or Brm antibody (4–6). The immunoprecipitates included the following gel lanes. Lane 1 & 4: 100 µg protein, 1 µg Brm antibody; lane 2 & 5: 200 µg protein, 1 µg Brm antibody; lane 3 & 6: 300 µg protein, 2 µg Brm antibody. C] PSF. As PSF apears in two molecular weights ∼100 KD and ∼49 KD (a proteolytic cleavage product [33], [34], we opted to use an immunoprecipitation kit (Pierce) that separates most antibodies used from the antigen that is then subjected to futher protein separation and immunodetection. Mouse cerebellar nuclear-enriched extracts (500 µg) from 8-month-old C57BL/129 male mice were subjected to immunoprecipitation with either 10 µg monoclonal PSF antibody (Sigma), (lanes 1) or 10 µg ADNP antibody (Bethyl, lane 2), followed by western blot analysis (10% polyacrylamide gel) with PSF antibody (1:2000 dilution; Sigma). As a control (lane 3), 500 µg of nuclear-enriched fraction from the thalamus and subthalamic nuclei were immunoprecipitated with 10 µg PSF antibody and subjected to ADNP western blot analysis (1:500 dilution; Bethyl, lane 3). D] Negative controls. Mouse nuclear protein extracts from the thalamus and subthalamic nucleus of 11-month-old C57BL/129 male mice (20 µg) were subjected only to western blot analysis to verify antibody binding (lane 1), the same nuclear-enriched extracts (500 µg) were further subjected to immunoprecipitation in absence of antibody (lane 2), for ADNP and Brm, western analysis was performed as in Fig. 5B PSF antibody (1:2000 dilution; Sigma) and ADNP antibody (1:500 dilution; Bethyl, lane 2). Mouse cerebellar nuclear-enriched extracts, (20 µg, lane 1, and 500 µg, lane 2) were further subjected to immunoprecipitation in absence of antibody, followed by western analysis with Brm antibody (1:1000 dilution; Sigma, as in Fig. 5C). Gel electrophoresis and extracts were as in 5B and C). E, F] Comparison between ADNP+/+ and ADNP+/- mice for Brm and PSF, ADNP interactions, respectively. Immunoprecipitation experiments were carried out as described in the materials and methods section. Western blot analysis was performed on the following samples: 1,5 Positive control (20 µg cortical lysate). 2,6 Negative control (IP without ADNP antibody). 3,7 First elution from the antibody affinity binding and specific antibody detection (as marked on the figure). 4,8 Second elution from the antibody affinity binding and specific antibody detection (as marked). 8% polyacrylamide gels were used for ADNP and Brm and 10% polyacrylamide was used for PSF. G] A suggested model of interaction.

The cerebellum does not express the transgene tau 4R, hence we hypothesized that ADNP levels will not change as a consequence of Tau 4R expression in other brain areas. Nuclear ADNP/actin ratios in the cerebellum were thus quantified by western blot analysis with ADNP and actin specific antibodies. Results indicated a significant increase in ADNP/actin ratio at 3 months of age in both Tau-Tg and non-Tg mice, followed by a decrease at 5.5 and 9 months of age (Fig. 4A and B). Factorial ANOVA analysis showed no interaction between transgenic condition and age (F(3,34) = 0.13055, *p* = 0.94126) and no main effect for the transgenic condition (F(1,34) = 0.01784, *p* = 0.89454), but found a main effect for age (F(3,34) =  16.996, *p* = 0.00001) (Fig. 5B). Tukey HSD post-hoc test for the age effect revealed a difference in nuclear ADNP amount between the 1- and 9-month old mouse groups (*p* = 0.0199) and between the 3- and 1- (*p* = 0.0002), 5.5- (*p* = 0.0004) and 9-month old (*p* = 0.0007) mouse groups.

### ADNP interaction with Brm and PSF, possible mechanism

Given the increased expression of ADNP and tau 3R in the presence of mutated tau 4R and the association of ADNP with the SWI/SNF and Brahma Related Gene 1 (Brg1), [10], it was of interest to test if ADNP interacts with Brahma (Brm), another member of the SWI/SNF complex and a known protein factor associated with alternative splicing [11]. Brm showed extensive expression in one-month-old Tau-Tg (Fig. 5A), correlating with the relatively high ADNP levels (please note that to avoid over exposure at 1-month, the 3-month levels are barely detectable). The increased ADNP protein levels in 1-month-old Tau-Tg mice also correlated with increased ADNP mRNA levels showing an RQ value of 9.422±0.921 compared to Tau-Tg 3-month-old 2.788±0.407 and to non-Tg 1-month-old 2.79±0.281 (*p*<0.001, see also Fig. 1).

Immunoprecipitation using Brm-specific antibodies identified both ADNP and Brm-like immunereactivity in the pellets, suggesting a direct interaction between ADNP and Brm. Increasing the amount of input brain extract as well as the amount of antibodies resulted in increased precipitation of both Brm and ADNP (Fig. 5B).

We have next investigated whether ADNP interacts with another factor recently shown to suppress tau exon 10 inclusion by interacting with a stem-loop structure downstream of the tau gene exon 10 [31], and previously shown to interact with the SWI/SNF-like complex [32]. As PSF apears in two molecular weights ∼100 KD and ∼49 KD [a proteolytic cleavage product [33], [34]], we used an immunoprecipitation kit (Pierce) which involved capturing theimmunoprecipitating antibody to protein A/G agarose resin and covalently immobilizing it by crosslinking with disuccinmidyl suberate (DSS), (avoiding general antibody recognition at ∼50KD as well). Results showed that the PSF antibodies detected the two expected protein bands in mouse cerebellar nuclear-enriched extracts (Fig. 5C, lane 1) and the same ∼49KD band was also detected following immunoprecipitation with the ADNP antibody, suggesting co-immunoprecipitation (Fig. 5C, lane 2). Previously, the 49 KD protein was described as a fragment constituting the N-terminal, protease-resistant half of the splicing factor PSF. Proteolytic degradation of PSF specifically occurs in intact cells and this process is enhanced upon cell lysis [34]. As a control (Fig 5C, lane 3), immunoprecipitation with PSF antibody (of nuclear enriched thalamic and subthalamic brain fractions) was performed and subjected to ADNP western blot analysis, ADNP-like immunoreactivity was detected at ∼120KD (Bethyl antibody, Fig. 5C, lane 3).

The apparent size differences between the ADNP immunoreactive bands interacting with PSF and Brm, are suggested to be trivial resulting from different experimental conditions during gel loading with reducing condition in the case of Brm immunoprecipitation, and Co-IP kit conditions with non-reducing gel loading condition in the PSF experiment (please see materials and methods). An additional negative control included immunprecipitation (IP) without the primary antibody (Fig. 5D) with results showing high specificity in terms of the immunoprecipitation. It should be noticed that all antibodies reconized some additional bands except those that are in the expected protein size (denoted on the figure), this could be a results of paucity of antigen in the brain region/age used in this instance (thalamus and subthalamic nuclei and the cerebelum of 8-month-old mice), protein degedation or not specific interactions.

To get an in depth answer for the ADNP-tau splicing regulation question, we have also looked for ADNP-PSF and ADNP-Brm interaction in the cerebral cortex of 3-month-old ADNP+/+ and ADNP+/− male mice (similar to the female mice used in Fig. 3C, in which the ADNP+/− showed a trend for an increase in tau 4R expression compared to ADNP+/+ at 2 months of age). Western blot analysis (Fig. 5E, 10% polyacrylamide, F, 8% polyacrylamise) indicated: 1] When looking at total protein extract, ADNP expression (protein band) was increased in the ADNP+/+ mice, compared to the ADNP+/− (150 kDa, lanes 1 and 4 in bottom panels of Fig. 5E and F), as expected [12]. Lower molecular weight bands were more abundant in the 8% polyacrylamide gel upon longer development of the immunoreaction. 2] Lanes 2 and 6 using immunoprecipitation without the specific respective antibody, did not show any protein band (as seen in Fig. 5D]. Immunoprecipitation with Brm antibodies showed some interaction with ADNP-like immunoreactivite 150 kDa band in the ADNP+/+ mice and to a much lesser extent in the ADNP+/− mice (Fig. 5E, lower panel, lanes 3 and 4 – antibody affinity binding and two consecutive elutions, for ADNP+/− compared to the respective lanes 7 and 8 for the ADNP+/+ mice). The reciprocal interactions, immunoprecipitation with ADNP antibodies and western blot with Brm antibodies did not show a distinct band at the expeced molecular weight of 230 kDa, but a smear which was slightly increased in the ADNP+/+ mice (Fig. 5E upper panel, lanes 7 and 8).

PSF immunoprecipiation and western blot analysis (8% polyacrylamide) followed the same gel loading pattern as for Brm (Fig. 5F). PSF and ADNP coimmunoprecipitated with either ADNP or PSF antiboy, and while PSF contect did not change in these 3-month-old ADNP mice (comparing ADNP+/+ to ADNP+/−), PSF-ADNP interaction was apparently decreased in the ADNP+/− mice. The immunoprecipitating ADNP-like band ∼50 kDa is a subject of future research.

## Discussion and Conclusions

The results in the current manuscript associated, for the first time, between transgenic over expression of mutated 4R tau (FTD model) and increased ADNP and tau 3R expression. The increased ADNP (mRNA and protein) occurring at the initial “disease” and decreasing thereafter was brain site specific (cerebral cortex, where the transgene is expressed, and not the cerebellum, where the transgene is not expressed). Shutting down the mutated tau transgene at 3 months of age resulted in normalization of both ADNP and tau 3R expression in the cerebral cortex.

The rTg(tauP301L)4510 mouse used here, expresses high levels of the human 4R tau with the P301L mutation (4R0N) in the neocortex and the hippocampus. While in the cerebral cortex, age-dependent differences in ADNP expression were observed between tau-Tg and non-Tg mice, no differences were found in the cerebellum. This finding suggests that ADNP expression might be regulated by Tg tau expression, and therefore differentiating between Tg and non-Tg mice only in parts of the brain that express high levels of transgenic tau 4R.

ADNP is highly expressed in the developing fetal brain, decreasing thereafter [7]. In fetal/juvenile brains from humans and rodents, the predominant spliced tau transcript does not contain exon 10, yielding the tau 3R protein. However, exon 10+ tau mRNA increases with age. In normal adult humans, there is a 1∶1 ratio of exon 10+ (4R) and exon 10− (3R) tau. In normal adult mice, the brain is enriched in 4R tau. It has been previously shown that the rTg4510 mice exhibit slower tau 3R turnover in response to human 4R tau over-expression, and increased tau 3R levels at 1 month and 3 month, as compared with non-transgenic mice [26]. We have now repeated and extended those studies to show a parallelism with ADNP expression and tau 3R expression. Thus, in our experiments, both tau 3R mRNA and protein expression in the cerebral cortex of 3-month-old mice were found to be significantly higher in rTg4510 Tau-Tg mice in comparison to control mice, in a brain region (cerebral cortex) that is specifically expressing the transgene. This might indicate that endogenous tau 3R expression is associated with modulation of ADNP expression, alongside with the expression of the transgenic human 4R tau.

ADNP expression is correlated with the need for brain protection [4], [5]. The initial increase in ADNP in the young Tau-Tg mouse may therefore indicate a compensatory mechanism, operated as an outcome of human transgenic 4R tau over expression, as well as increased levels of endogenous 3R hyperphosphorylated tau in the cerebral cortex [26]. The increase in 3R tau alongside with ADNP, may also act as a compensatory mechanism, as 3R tau was shown to transiently accumulate in cell nuclei, bind DNA and provide protection against DNA damage [19]. However, it is hypothesized that the ADNP increase at the onset of the disease progression is not sufficient to provide protection in the face of continuous neurodegenerative tau-related process and age-related decreases. The increased neurodegeneration at an older age coupled to ADNP reduction implicates loss in ADNP–expressing neurons and glia, which is suggested to be related to loss in neuroprotection. A similar pattern of increased ADNP expression in the young compared to old transgenic was observed in an Alzheimer's disease mouse model, the PS1xAPP [PS1(M146L) x APP(751SL)] transgenic mice, which was specific to the hippocampus, but not the cerebellum [35].

Interestingly, in the cerebellum, there was a significant increase in ADNP-like immunoreactivity in 3-month-old mice which might be indicative of the delayed development of the cerebellum in comparison to the cerebral cortex. These age-dependent differences are in agreement with the multiple gene expression changes observed in the developing hippocampus [36]. Given that ADNP is a key regulator of gene expression associated with neuronal maturation [7], [8] and given that it is also part of the SWI/SNF complex which may be associated with neuronal specification [10], [37], these results indicate a potential change in neuronal specification as a consequence of tau mutation and ADNP deregulation in the cerebral cortex.

Regarding the SWI/SNF chromatin remodeling complex, in-gel protein digests followed by mass spectrometry identified several components of the SWI/SNF complex as co-immunoprecipitating with ADNP including, Brg1 (Brahma Related Gene 1), and the Brg1-associated factors (BAFs), BAF250a, and BAF170, all components of the SWI/SNF complex [10]. Brg1 and BAF 170 were also found to be associated nuclear receptor co-repressor (N-CoR) that is in turn associated with the splicing machinery, suggesting coordination of pre-mRNA splicing with chromatin remodeling events and transcriptional regulation [38]. In this respect, Brm (Brahma) was found to be a regulator of alternative splicing favoring axon inclusion, which may be associated with the observed high expression in the transgenic mice favoring the tau 4R expression (inclusion of exon 10) at a young age. Furthermore, in tau knockout mice, exhibiting delayed axogenesis, the expression of Brg1-associated factor, BAF-57, a protein involved in the repression of neuron specific genes, showed a marked over expression [39]. Importantly, our current results show for the first time a direct interaction between Brm and an immunoreactive ADNP band. Additional studies have shown that Brm and Brg1 interact with the DBHS (Drosophila behavior, human splicing) family protein, p54^nrb^ to regulate splicing. PSF (polypyrimidine tract-binding protein-associated splicing factor), another DBHS family protein known to directly bind p54^nrb^, was also found to associate with the SWI/SNF-like complex [32]. Most recent studies showed a direct association of PSF with tau splicing indicating that PSF suppresses tau exon 10 inclusion by interacting with a stem-loop structure downstream of exon 10 [31]. This is of main interest as we are now showing comimmunoprecipitation of PSF and an immunoreactive ADNP band. Further studies are warranted to evaluate potential brain region differences in ADNP-PSF/Brm interaction as well as age-dependency. Regardless, our results suggest that ADNP may take part in the regulation of tau splicing (Fig. 5G) and together with PSF suppress tau-exon-10 inclusion, which correlates with its expression patterns and the reduction in tau 3R in aging rodents compared to young rodents as well as to trending increases in tau 4R in young ADNP-deficient mice. Interestingly, PSF regulation and the regulation of tau splice variant expression have both been associated with learning and memory [40]. ADNP-deficiency has been associated with cognitive and social deficits as well as tauopathy and brain cell death in mice [12] and mental retardation [41] as well as autism [42], [43] in men. Further studies have shown deregulation of ADNP content in schizophrenia [44], multiple sclerosis [45] and Alzheimer's disease [46] in men, suggesting a central role in neuroprotection, which is now extended to regulation tau expression, paving the path to better understanding of brain degeneration/protection.

It is a question for further research to analyze if tau protein stability is modulated by ADNP loss of function. In this respect, 1] ADNP is highly expressed in the developing embryo, showing a peak of expression at E9–14 (a time of high 3R tau expression) [7]. 2] The ADNP snippet peptide, NAP (NAPVSIPQ) enhances tau-microtubule interaction [47], [48], which should protect against tau degradation. 3] In Alzheimer's patient serum samples, ADNP was the only protein that showed a significant reduction as measured by in depth proteomics [46]. In this respect, our current results showed reductions in ADNP expression with age which may be augmented in the Tg-Tau model (Fig. 1).

A recent publication has shown that PSF interacts with peroxisome proliferator-activated receptor gamma (PPARγ), a nuclear receptor that plays an essential role in cell proliferation, apoptosis, and inflammation [49], serving as a therapeutic target in Alzheimer's disease [50], [51]. PSF knockdown induced apoptosis via activation of caspase-3, and it was suggested that this is due to the disrupted interaction with PPARγ [49]. Our original studies have shown that some of the promoters of the ∼250 genes found to be up-regulated by ADNP-deficiency were also found to be enriched in regulatory elements that interact with PPARγ [8]. Furthermore, PSF (also known as SFPQ, splicing factor, proline- and glutamine-rich) is depleted from the nucleus and accumulates in the cytoplasm in Alzheimer's and Pick's disease, in brain areas affected by tau pathology. This cellular localization is mediated by tau over-expression [21]. Together, these findings associate PSF and ADNP beyond the regulation of tau splicing, to co-regulation of gene expression. Interestingly, ADNP can suppress its own gene expression [8], [52], suggesting an internal control mechanism.

ADNP deficiency can be ameliorated in part by compensatory treatment with the ADNP fragment, the drug candidate davuentide (NAP), that stabilizes microtubules and inhibits tau pathology while protecting cognitive [12] as well as motor functions [53], by protecting microtubule-dependent axonal transport [48], [53]. As the current studies concentrate on ADNP, it is of interest to note that the increase of ADNP at the time of disease onset suggests a potential protective effect for ADNP enhancement/replacement therapy at disease onset and later on.
